# Cancer care in conflict-affected regions: a scoping review of service delivery challenges, healthcare system adaptations, and policy implications

**DOI:** 10.1186/s13031-026-00816-y

**Published:** 2026-06-19

**Authors:** Omar Al-Qaisi, Shend Kryeziu, Lorent Sijarina, Patricia Tai, Melisa Stublla, Edward Yu, Maha Subih, Mohammed Dibas

**Affiliations:** 1 Nursing Department, Al-Zaytoonah University, Airport Street, Amman, Jordan; 2 Faculty of Medicine, University of Prishtina, Prishtina, 10000 Kosovo; 3 Department of Oncology, University of Saskatchewan, Saskatoon, SK S7N 5A2 Canada; 4 Department of Oncology, Western University, London, ON N6A 3K7 Canada; 5 Department of Medicine, Faculty of Medicine and Health Sciences, An-Najah National University, Nablus, P400 Palestine

**Keywords:** War, Conflict, Oncology, Cancer, Conflict-affected settings, Humanitarian health, Healthcare

## Abstract

**Background:**

Armed conflicts severely disrupt cancer care delivery, destroying infrastructure and limiting access to essential diagnostics and treatments. Evidence on service challenges, adaptations, and policy implications remains fragmented.

**Methods:**

This scoping review of four databases (Scopus, PubMed, ScienceDirect, and CINAHL) was conducted for original peer-reviewed studies published between January 2020 and August 2025. The search focused on the effect of wars on cancer service delivery in conflict zones, and its impact on cancer care.

**Results:**

Wars in conflict-affected countries like Palestine, Sudan, Syria, Iraq, Ukraine, and Afghanistan caused widespread destruction of cancer infrastructure, unsafe hospitals, and interruptions in chemotherapy, radiotherapy, and surgery. Countries faced shortages of specialized centers, technicians/radiologists, and medical records, alongside high costs and supply chain disruptions. Despite these challenges, healthcare systems demonstrated resilience through adaptations including Ukraine’s MedEvac program for EU treatment evacuation, cross-border referrals (Iraq→Lebanon, Afghanistan→Pakistan), telemedicine, mobile diagnostic units, and community-based care models. Policy responses emphasized international aid coordination, conflict-sensitive health planning, and supply chain restoration.

**Conclusion:**

Conflicts cause infrastructure destruction, workforce depletion/migration, treatment barriers/delays, drug supply disruptions, and psychosocial/economic impacts, which in turn create urgent policy/governance challenges. Although humanitarian aid may provide temporary relief, sustainable solutions require peace and global commitment, grounded in equity and the fundamental right to health.

**Supplementary Information:**

The online version contains supplementary material available at 10.1186/s13031-026-00816-y.

## Introduction

Although some wars have been ended, like in Syria and Gaza, armed conflict still continues; in addition, some wars, like in Ukraine and Sudan, have persisted despite international efforts to resolve these conflicts . Other areas like Yemen, Iraq, Lebanon, and Afghanistan still suffer now from the effect of wars. This left their healthcare infrastructure in ruins, with years of rebuilding ahead. Deeply concerned about cancer patients’ welfare amid disrupted services, the authors conducted this scoping review on service delivery challenges, system adaptations, and policy implications.

Cancer care in conflict-affected regions represents a critical intersection of humanitarian crisis and public health emergency, as over one billion people currently live in fragile, conflict-affected, and vulnerable settings. The systematic disruption of oncology services carries profound implications for cancer outcomes and health equity, demanding urgent attention from the global health community [1].

The global cancer burden has reached an unprecedented level, with an estimated 20 million new cancer cases and 9.7 million cancer-related deaths recorded in 2022 [2]. Conflict-affected regions experiencing disproportionally poor outcomes due to healthcare infrastructure destruction, healthcare professional displacement, and systematic barriers to access timely diagnosis and treatment [3]. Armed conflicts profoundly disrupt every stage of cancer care, diagnostic services are often delayed as imaging equipment is destroyed, laboratory services are interrupted, and skilled personnel flee conflict zones [4]. Additionally, follow-up care becomes nearly impossible when patients are displaced and medical records are lost [5, 6]. Healthcare systems in conflict zones show varying resilience through adaptations like cross-border referrals and community-based care models. These innovations provide vital lessons for improving cancer care in humanitarian crises [7]. Cancer care disruptions in these areas undermine health equity and human rights, violating the right to health under international law [8]. This necessitates the development of conflict-sensitive health policies in high-risk environments [9]. Recent innovations such as telemedicine, mobile diagnostic units and AI-powered tools bridge geographical and logistical barriers in cancer care delivery [10]. Similarly, community engagement and local capacity building have emerged as fundamental principles for sustainable cancer care in conflict-affected regions [11].

Despite the growing body of literature on cancer care disruptions in conflict-affected settings such as Ukraine and Syria, existing literature remains fragmented, focusing on single countries or general humanitarian health rather than cancer-specific service delivery. This scoping review investigates cancer care challenges in conflict zones, healthcare system adaptations, and policy implications for global health. Through systematic analysis of service delivery challenges and adaptive responses across different conflict settings, it aims to contribute evidence-based recommendations for strengthening cancer care systems in humanitarian emergencies and promoting equitable access to oncology services for the world most vulnerable populations.

## Method

Choosing between scoping and systematic reviews depends on the purpose of the review. A scoping review was more appropriate than a systematic review in this situation given the heterogeneity of study designs, exploratory mapping of evidence and limited high-quality data. A scoping review is useful in identifying knowledge gaps and exploring the health of a specific population.

This scoping review was conducted following a comprehensive approach. The search was conducted in four databases: Scopus, ScienceDirect, PubMed, and CINAHL (Cumulative Index to Nursing and Allied Health Literature). The search was done twice. Once the initial pool of literature was identified, the next step involved screening. Inclusion criteria were defined as articles (1) published between January 2020 and August 2025, (2) English language only, and (3) only original peer‑reviewed research articles that met all eligibility criteria (quantitative, qualitative, or mixed‑methods designs conducted among adult populations). Exclusion criteria included commentaries, letters to the editor, conference abstracts, theses, case reports, editorials, and non-peer-reviewed articles. All editorials were excluded except one that specifically addressed cancer care in Gaza post-October 7 due to the paucity of available literature on this topic.

### Search outcome

**Fig. 1:**
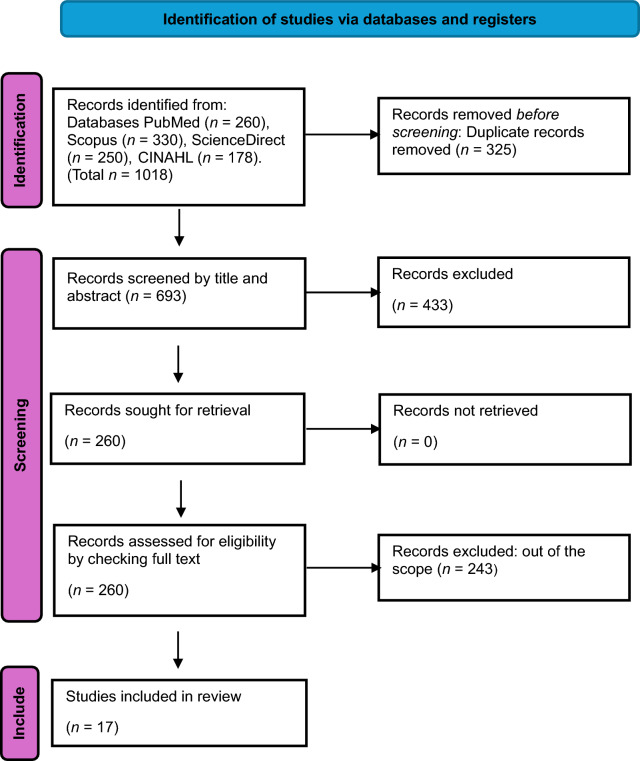
Preferred reporting items for systematic review and meta-analysis extension for scoping reviews (PRISMA-ScR) flow diagram of studies to include in this scoping review

Of the 1018 records identified from the databases, 325 duplicates were excluded, leaving 693 unique records for title and abstract screening. During this stage, 433 records were excluded for not meeting the inclusion criteria (e.g., not conducted in conflict‑affected regions, not focused on cancer care or service delivery, non‑original research, or non‑adult populations). The remaining 260 full‑text articles were assessed for eligibility, of which 243 were excluded as out of scope, resulting in 17 studies included in the final review (Fig. 1).

The keywords used for the search were "cancer care", "war", "conflict-affected regions", "service delivery", "healthcare system adaptations", "policy implications", "fragile states", "humanitarian crisis", and "healthcare access" by using different combinations of Boolean operators. Only original research articles which satisfied all inclusion criteria were included. Two independent reviewers screened titles/abstracts and later full-text articles. Discrepancies were resolved through consensus. The selection process is detailed in the PRISMA-ScR flow diagram (Fig. 1) Data were extracted manually using a standardized form, capturing: Author, Purpose, Settings, Sample size, Study design, Main findings (Table 1). A completed PRISMA-ScR reporting checklist is provided in Supplementary Table S1 in the Appendix. The search results presented in Fig. 1.

**Table 1:** Studies included in the final literature review

Author, year of publication	Purpose of the study	Country	Sample size	Design	Main findings
Hovhannisyan A (2024) [1]	To explore experiences and barriers faced by patients from Nagorno-Karabakh in accessing cancer care during conflict and blockade	Nagorno-Karabakh / Armenia	12 adult cancer patients + 12 cancer care professionals	Qualitative study	The blockade disrupted transport and access; free medication programs were paused; patients incurred high travel and accommodation costs; treatment delays and interruption were major problems.
Basha L (2024) [3]	To systematically assess available evidence on the burden of cancer and the status of oncology services in Syria during conflict	Syria	Not applicable	Systematic review	28 eligible studies identified (academic + grey literature). Conflict disrupted oncology services via staff shortages, chemotherapy and radiotherapy drug shortages, concentration of services in Damascus, inequities in access, and increased mortality and incidence in some regions.
Al-Ibraheem A (2022) [4]	To examine challenges & constraints in diagnosing cancer (especially imaging, pathology) in conflict-affected regions of the Middle East	Conflict-affected countries: Iraq, Syria, Yemen, Sudan	Not applicable	Narrative review	Conflict impedes diagnostic services (imaging, labs), causes delays in biopsy/pathology, forces patients to seek diagnosis abroad with high costs, leads to late-stage detection, exacerbates inequalities in access to care.
Ahmed Y (2023) [6]	To propose strategies and priorities for optimizing cancer care services in conflict zones / wartime settings	Conflict-affected zones (general & not limited to one country)	Not applicable	Perspective / proposal paper	The article outlines key priorities: needs assessment, treatment prioritization, secure drug supply chains, telemedicine, mobile clinics, cross-border collaboration, staff support, and care continuity. It urges multi-stakeholder coordination and technology integration to safeguard cancer care in war zones.
Skelton M (2020) [12]	To examine the experience of Iraqi cancer patients seeking treatment in Lebanon, including social and financial contexts under conflict.	Iraq/ Lebanon	60 participants (patients/ caregivers)	Mixed-methods (combining quantitative survey and qualitative interviews)	Patients faced very high treatment costs, financial hardship, and limited institutional support. Conflict exacerbated barriers to access, disrupted continuity of care, and increased emotional and financial distress among patients.
Caglevic C (2022) [13]	To assess how armed conflict (the war in Ukraine) impacts cancer patients — their access to treatment, continuity of care, psychosocial and economic effects.	Ukraine	Not applicable	Narrative review	The war severely disrupted oncology services, displaced patients, causes treatment delays and led to shortages of medications and medical staff. The authors call for international support and coordination to sustain cancer care during conflict.
AlWaheidi S (2020) [14]	To describe how the COVID-19 pandemic has created additional challenges for cancer patients living in Gaza, compounding pre-existing difficulties caused by blockade and limited healthcare resources.	Palestine (Gaza Strip)	Not applicable	Narrative review	Cancer patients in Gaza faced severe barriers to accessing care during COVID-19, including travel restrictions, treatment interruptions, shortages of medications, and lack of protective equipment. The pandemic further strained an already fragile oncology system under blockade conditions.
Mansour R (2024) [15]	To explore and map systemic barriers that hinder access to high-quality cancer care in Jordan, and propose solutions	Jordan	Not applicable	Narrative review	Weak infrastructure, workforce shortages and financial and insurance gaps as major barriers to cancer care, and recommended decentralization, improved training, telemedicine and policy reforms to enhance service delivery.
Van Hemelrijck M (2022) [16]	To assess and model the impact of the early conflict in Ukraine on cancer care needs of refugees, and to estimate how cancer cases may be distributed across host countries.	Ukraine & neighboring host countries	Not applicable	Strategic impact assessment / modeling study	Estimated that Ukraine had ~13,106 new cancer patients per month pre-conflict; estimated ~33,121 cancer cases among refugee populations (based on 7.5 million refugees). Poland is assessed as having relatively better capacity, while other neighboring countries (Hungary, Romania, Moldova, Slovakia) have variable capacity levels to absorb extra cancer care burden.
Mohsin K (2024) [17]	To identify and categorize the barriers to delivering and accessing cancer care in Iraq during conflict periods (1980–2017) using the HealthCare Access Barrier (HCAB) framework.	Iraq	Not applicable	Narrative review	The review categorized barriers into financial, structural, and cognitive. Financial barriers included lack of funding and affordability; structural barriers involved destroyed or inaccessible facilities, limited diagnostics, workforce shortages, and disrupted services; cognitive barriers included low awareness and poor health literacy. The conflict significantly exacerbated financial and structural barriers, worsening access and continuity of cancer care.
Mahmood S (2023) [18]	To understand the journey and outcomes of Afghan cancer patients who seek treatment in Pakistan due to conflict disruptions in Afghanistan	Afghanistan → Pakistan	6,370 patients	Retrospective observational study	Of the 6,370 patients treated since 1995: mean age ~ 40.7 years, 57% male; 56% presented at stage III/IV. 34% of adults achieved complete response, but over half were lost to follow-up. Children had somewhat better outcomes (43% complete response). The conflict and limited diagnostic infrastructure forced many patients to travel, contributed to treatment discontinuation, and worsened outcomes.
Skelton M (2023) [19]	To assess the impact of the ISIL conflict on cancer care access, infrastructure, and service delivery across five affected Iraqi provinces.	Iraq (Nineveh, Kirkuk, Salah al-Din, Anbar, Diyala)	3,855 cancer cases reviewed between 2014–2017	Retro-spective cross-sectional / observational study	Conflict severely disrupted oncology services — destruction of health facilities, shortage of staff and chemotherapy, migration of oncologists, and interruption of radiotherapy. There was an increase in late-stage (III–IV) presentations and uneven recovery across provinces. The study calls for decentralization and international assistance to rebuild cancer care systems.
Hanafi I (2024) [20]	To investigate how the Syrian war affected the diagnosis, stage, and outcomes of lung cancer between 2011 and 2018.	Syria	1,048 patients diagnosed with lung cancer at Al-Mouwasat University Hospital (2011–2018)	Retrospective observational study	The war caused diagnostic and treatment delays, limited access to imaging and pathology, and an increase in late-stage (III–IV) presentations. Only 22 % of patients received curative-intent treatment; survival rates declined markedly during conflict years. The study underscores the long-term negative impact of armed conflict on cancer outcomes.
Huivaniuk I (2024) [21]	To assess the impact and structure of the “MedEvac” program facilitating evacuation of Ukrainian cancer patients to EU institutions for ongoing care during prolonged conflict.	Ukraine → European Union (during Russian invasion)	281 evacuated patients (from 639 applications)	Retrospective cohort / observational study	Of 639 applications, 339 had sufficient data; 281 (82.9 %) were evacuated. Median age 47 years. Most patients (94 %) were newly diagnosed. The majority (81.9 %) evacuated for systemic therapy. Good performance status (ECOG 0-2) was strongly associated with the likelihood of evacuation. The program is a viable model for managing continuity of cancer care under conflict circumstances.
Nashwan AJ (2023) [22]	To explore how cancer care was impacted during the COVID-19 pandemic in conflict-affected settings	Gaza Strip, Palestine	Not applicable	Editorial	The study emphasizes the severe shortage of chemotherapy drugs, restricted patient movement for treatment abroad, and limited diagnostic capacity due to the blockade. It also highlights the psychological distress among oncology staff and patients, urging global health agencies to support sustainable cancer care in Gaza.
Ahmed I (2024) [23]	To document how the ongoing armed conflict in Sudan is leading to collapse of cancer care services and propose strategic responses	Sudan	Not applicable	Narrative analysis / commentary	The conflict has severely damaged cancer infrastructure (e.g., Khartoum Oncology Hospital nonfunctional), disrupted supply chains, displaced health workers, and forced patients to travel far or forego treatment. The article calls for urgent international support to rebuild services and protect oncology patients.
Abasher MM (2024) [24]	To analyze the impact of war on the availability and accessibility of chemotherapy drugs for cancer patients in Sudan	Sudan (Port Sudan Oncology Center)	File records from Port Sudan Oncology Center (April–August 2023	Quantitative retrospective analysis	Fluctuating & inconsistent chemotherapy drug supplies: some essential drugs showed complete absence or intermittent availability. These shortages severely compromised treatment continuity, increased morbidity & mortality, & caused significant socioeconomic and psychological burdens on patients. It calls for resilient supply chain systems, international collaboration, and infrastructure strengthening to ensure continuous access to cancer drugs in Sudan and other conflict zones.

COVID-19: coronavirus disease 2019 ISIL: Islamic State of Iraq and the Levant.

Articles unrelated to cancer care in conflict-affected regions were excluded after reviewing titles and abstracts. All articles addressing cancer service delivery challenges, healthcare system adaptations, and policy implications in conflict settings that resulted from the search were reviewed.

### Data extraction

The software EndNote was used for data extraction. The process of selecting the final 17 studies is outlined in Fig. 1, and the studies are listed in Table( 1) [1, 3, 4, 6, 12–24].

## Results

### Cancer prevalence and epidemiological profile in conflict-affected populations

Table 2 presents the prevalence of cancer and key epidemiological indicators across conflict-affected populations identified in this scoping review. Among Ukrainian refugees, the total estimated cancer cases (combining prevalent and incident) reached 33,121 individuals, with Poland bearing the largest burden (19,284 cases), followed by Romania (5,281), Slovakia (2,421), Moldova (2,651), and Hungary (3,484). The total new cancer patients per month in pre-conflict Ukraine was estimated at 13,106, comprising both male and female populations [16].

**Table 2:** Cancer prevalence and epidemiological indicators that are available in conflict-affected populations

**Region/Population**	**Time period**	**Sample size**	**Cancer incidence rate**	**Common cancer types**	**Stage at presentation**	**Mortality/survival data**	**Reference**
Ukraine (refugees)	2022-2023	33,121 estimated	13,106 new cases/month	Breast, Colorectal, Lung	N/R	N/R	Van Hemelrijck, 2022 [16]
Syria (national)	2022	N/A	ASR 135.7/100,000	Breast, Colorectal, Lung	60-80% stage IV	ASR mortality 89.6/100,000	Basha, 2024 [3]
Syria (lung cancer cohort)	2011-2018	5,160	N/A	Lung	63.6% with metastases	1-yr survival: 13.1%; 5-yr: 0.1%	Hanafi, 2024 [20]
Syria (Northwest)	2020	379	N/A	Breast (38.3% F), Bladder (15.7% M)	64.7% breast cancer stage III+	N/R	Atassi, 2022 [15]
Iraq (national)	2020	31,692	CIR 78.93/100,000	Breast, Lung, Colorectal	N/R	N/R	Mohsin, 2024 [17]
Iraq→Lebanon	2013-2016	1,284 registries	N/A	Varied	N/R	90% financial burden	Skelton, 2020 [12]
Afghanistan→Pakistan	1995-2022	6,370	N/A	Esophageal (20.2%), Gastric (10.1%), Breast (9%).	56% stage III-IV	1-yr survival <50%	Mahmood, 2023 [18]
Nagorno Karabakh→Armenia	2023	24 patients interviewed	N/A	Varied	N/R	N/R	Hovhannisyan, 2024 [2]
Gaza	2021-2023	2000 patients	ASR 91.3/100,000	N/R	N/R	610 deaths in 2020	Nashwan AJ, 2023 [1]
Sudan	Up to 2022	N/A	Rising sharply	N/R	Advanced stages due to delays	Poor access worsens outcomes	Al-Ibraheem A (2022) [4]

ASR: Age-Standardized Rate; CIR: Crude Incidence Rate; F: Female; M: Male; N/A = Not applicable; N/R: Not Reported; yr: year.

In *Syria*, the age-standardized incidence rate (ASR) for all cancers reached 135.7 per 100,000 population in 2022, with an ASR mortality of 89.6 per 100,000, representing a 31.7% increase in incidence and 40% increase in mortality compared to 2015 estimates [3, 25]. Breast cancer emerged as the most common malignancy among Syrian women, with ASR incidence rising from 52.5 per 100,000 in 2012 to 67.3 per 100,000 in 2018, before declining to approximately 46 per 100,000 in 2022 [26]. Northwest Syria data from 2020 documented 379 cancer cases diagnosed during active conflict, with breast cancer accounting for 38.3% of cancers in women and bladder cancer representing 15.7% in men. Notably, 64.7% of breast cancer specimens were stage III or above at diagnosis [5]. *Iraq* demonstrated a crude incidence rate (CIR) of 78.93 per 100,000 for the overall country in 2020, with conflict-affected provinces showing variable CIR: Diyala (74.35/100,000), Anbar (65/100,000), Salah al-Din (64.52/100,000), Nineveh (63.94/100,000), and Kirkuk (56.8/100,000) [17]. Among Iraqi patients seeking cross-border care in Lebanon, a registry analysis identified 1,284 patients receiving treatment between 2013-2016, with 90% experiencing financial burden [12, 17].

Lung cancer data from Syria revealed that among 5,160 patients diagnosed during 2011-2018, metastases were present in 63.6% at diagnosis, with bone (39%) and liver (21%) being the most common metastatic sites. The 1-year survival rate was 13.1%, and the 5-year survival rate reached only 0.1% [20]. Among Afghan patients receiving care in Pakistan, 6,370 individuals were registered between 1995-2022, with 56% presenting with stage III or IV disease. Upper gastrointestinal malignancies, including esophageal (20.2%) and gastric cancer (10.1%), were the most common cancers among adults [18].

### Infrastructure destruction and service disruption

Armed conflicts across multiple settings resulted in widespread destruction of cancer care infrastructure, severely limiting diagnostic and therapeutic capacity. In *Syria*, the Al-Kindi Hospital in Aleppo, formerly the largest hospital in northern Syria, was entirely destroyed during the conflict, compelling patients to converge on Al-Beiruni Cancer Center in Damascus for specialized care [3, 26]. The Syrian healthcare system experienced concentration of services in government-controlled Damascus, where 89.6% of cancer consultations occurred, while entire regions like East Aleppo were left without oncologists or pathologists to serve 500,000 people [3].

Iraq’s conflict-affected provinces exhibited heterogeneous patterns of infrastructure damage. In Nineveh, the complete destruction of Mosul’s oncology infrastructure during the liberation campaign from ISIL necessitated reconstruction from ground zero, with oncologists working in makeshift structures years after conflict cessation [19]. Anbar province witnessed the suspension of all oncology services when ISIL occupied areas containing the oncology department, forcing the complete evacuation of medical personnel [19]. Ukraine’s experience showed rapid degradation of healthcare delivery, with the National Cancer Institute in Kyiv, which had served 400 patients daily with 600 beds and over 1,000 employees, facing severe operational constraints within weeks of conflict onset [13].

In Gaza and Sudan, cancer infrastructure collapsed rapidly under airstrikes and bombardment: Gaza’s sole cancer hospital (Turkish Palestinian Friendship Hospital) was hit on Oct 30, 2023, then forced to shut on Nov 3, killing 4 patients outright and displacing 70 to inadequate Khan Yunis facilities [22]. Sudan’s Khartoum cancer centers were bombed and abandoned, obliterating radiotherapy, chemotherapy, and diagnostic capacity for large parts of the population, which pushed patients to under-resourced peripheral hospitals or out of the country, and likely increased cancer-related mortality, although precise survival data are lacking [23, 24].

Afghanistan’s long-standing conflict has obliterated cancer care infrastructure, leaving the country without functional oncology centers, radiotherapy units, or advanced diagnostic facilities, and forcing patients to cross into Pakistan, where most arrive with late-stage diseases when curative options are limited [18]. Yemen, meanwhile, entered its wars with an already fragile cancer care baseline that conflicts rapidly dismantled, as severe shortages of specialized personnel and limited access to essential cancer treatments compounded the collapse of diagnostic and therapeutic capacity, driving patients abroad in search of care [4].

Diagnostic capacity deterioration emerged as a critical consequence across different conflict settings. Yemen possessed only 5 gamma cameras (0.2 units per million people), predominantly non-functional due to manpower and radiopharmaceutical shortages [4]. Iraq’s conflict-affected provinces lacked functional radiotherapy equipment entirely, with zero linac machines available in Nineveh, Anbar, Kirkuk, Salah al-Din, and Diyala as of 2022, despite 34 functional linacs existing nationally [19]. Syrian nuclear medicine physicians declined from 11 to 5, radiologists from 98 to 73, and diagnostic services became increasingly concentrated in Damascus and Lattakia [3].

### Healthcare workforce depletion and migration

Conflict-induced exodus of specialized oncology professionals constituted a fundamental barrier to sustained cancer care delivery. Syria witnessed approximately 70% of healthcare workers fleeing the country, with oncology experiencing particularly acute shortages [3]. Northwest Syria reported only 35 oncologists serving the entire population in 2023, with inadequate staffing cited as a primary factor contributing to poor cancer outcomes [3]. Iraq experienced mass exodus of approximately 20,000 physicians since the 1990s, with an estimated 320 doctors killed since 2003, leaving only 52 clinical oncologists and 76 radiation oncologists to serve a population requiring at least 350 oncologists [17, 19].

The shortage of specialized workforce extended beyond physicians to encompass nursing and allied health professionals. Jordan, while not directly experiencing conflict but absorbing Syrian refugees, reported significant oncology nursing shortages that strained existing staff and compromised patient care quality, with increasing nurse-patient ratios directly impacting patient experiences and outcomes [15]. Afghanistan’s healthcare system degradation following decades of conflict resulted in complete absence of radiation therapy facilities domestically, with no radiation oncologists available within the country [18].

Geographic maldistribution of remaining healthcare workers exacerbated access barriers. Damascus maintained the best physician-to-population ratio with 20 oncologists for a population exceeding one million, while other Syrian regions had far fewer specialists [3]. Sudan’s Darfur region, serving approximately 9 million people, had only one area with radiology services, one pathology laboratory, and no nuclear medicine services [4]. This urban-rural divide in healthcare professional distribution forced patients to undertake dangerous long-distance travel or forgo treatment entirely.

### Treatment access barriers and delays

Patients in conflict-affected regions faced multifaceted barriers to accessing timely cancer treatment, resulting in advanced-stage presentations and compromised outcomes. Military blockades and checkpoints constituted immediate physical barriers to care access. Nagorno-Karabakh patients described the December 2022 Azerbaijani military blockade of the Lachin Corridor as creating insurmountable obstacles, with patients requiring Red Cross coordination for medical evacuation, a process taking up to 1.5 months to establish [1]. Syrian patients from besieged areas experienced difficulties, with travel restrictions and security checkpoints preventing movement between regions for treatment [3, 20].

Financial barriers intensified during conflict periods. Iraqi cross-border cancer patients demonstrated that 90% experienced financial burden, with approximately one-third selling possessions including homes to cover treatment and travel expenses [12]. Monthly cancer care costs in Syria reached $1,000-1,500 while median Syrian monthly salary fell to $150 in 2017, rendering treatment financially prohibitive [3]. Northwest Syria’s population, with 90% living below poverty line, faced additional challenges as cancer care was classified as tertiary healthcare and excluded from donor funding, requiring NGOs to independently fundraise for cancer services [3].

Geographic distance and transportation challenges compounded access difficulties. Afghan patients traveled up to 2,000 kilometers or 26 hours to reach cancer treatment facilities in Pakistan [18]. Iraqi patients from Mosul and surrounding provinces depended on transportation to Baghdad, Basra, Karbala, Erbil, and Najaf, with long distances and transport costs contributing to lower service utilization [19]. Nagorno-Karabakh patients described challenges finding transport and traveling long distances to Armenia, with treatment burden particularly severe for patients requiring repeated weekly tests who needed multiple dangerous journeys [1].

Late-stage diagnosis emerged as a consistent pattern across conflict settings. Syrian lung cancer patients presented with metastases in 60-80% of yearly diagnoses regardless of temporal-geographical region, with 63.6% having metastases at diagnosis [20]. Northwest Syrian breast cancer data showed 64.7% of specimens at stage III or above, while Damascus hospital data revealed 61% of breast cancer patients presenting at stage III or higher [3, 5]. Afghan patients in Pakistan demonstrated 56% presenting with stage III or IV disease, with upper gastrointestinal malignancies particularly advanced at presentation [18]. Syrian refugees in Jordan presented predominantly with locally advanced or metastatic breast cancer, with over one-third experiencing deviations from standard treatment guidelines and worse outcomes compared to the general Jordanian population [15].

### Drug supply chain disruptions

Pharmaceutical supply chain disruptions severely compromised cancer treatment continuity across conflict settings. Sudan’s analysis of chemotherapy drug accessibility revealed fluctuating availability patterns during distinct conflict periods, with medications exhibiting availability ranging from consistent supplies to complete absence [24]. Iraq experienced the 13-year economic sanctions (1990-2003) that severely restricted imports of chemotherapy drugs and medical equipment, substantially hampering diagnostic and therapeutic capacity [17].

Yemen’s conflict rendered the country unable to produce radioiodine domestically, creating complete dependence on international suppliers amid trade disruptions [4]. Syria experienced medication shortages in Damascus forcing regimen modifications, with essential cancer medicines sometimes available but experiencing shortages typically once or twice per month, often causing treatment delays of several months [3]. Advanced cancer medicines such as targeted therapy showed limited availability across all Syrian regions including Damascus [3].

Paradoxical patterns emerged in some settings. Kirkuk, Iraq, experienced its pharmaceutical supply peak during the ISIL conflict period (2014-2017) when the Ministry of Health transferred large portions of pharmaceutical stocks designated for Nineveh, Salah al-Din, and Diyala provinces to Kirkuk to account for displaced patient burden. Important chemotherapy agents including Herceptin, Avastin, Cisplatin, and Carboplatin remained reliably available throughout the ISIL period—a stark contrast to pre-conflict scarcity and post-conflict regression [19].

Nagorno-Karabakh’s blockade halted decentralized medication supply policies, with patients unable to transport refrigerated chemotherapy medications across Azerbaijani checkpoints due to lack of trusted mediators and concerns about proper storage and handling [1]. Northwest Syria reported that patients requiring advanced treatments including radiotherapy had to be referred outside Syria, with waiting times between referral and treatment in Turkey ranging from one to six months, a process that stopped entirely following the February 2023 earthquake [3].

### Healthcare system adaptations and resilience

Despite overwhelming challenges, healthcare systems demonstrated adaptive responses to maintain cancer care delivery. Mobile health units emerged as critical adaptation strategies across multiple settings. The King Hussein Cancer Center implemented mobile clinics visiting remote and impoverished areas of Jordan, providing free initial detection services for common cancers and screening more than 5,000 patients annually in communities with poverty and facility shortages [15]. Afghanistan’s Comprehensive Cancer Care Center operated mobile cancer screening units to reach rural communities [18].

Telemedicine and tele oncology platforms facilitated continuity of care despite geographic and security barriers. The Syrian American Medical Society (SAMS) implemented telemedicine to provide oncology consultations to patients in conflict-affected Syrian areas, enabling remote consultations, diagnostics, and treatment planning while serving educational purposes for capacity building of local healthcare workers [3]. Jordan developed telemedicine capabilities to support refugee populations, though implementation challenges persisted due to inadequate information technology infrastructure including absence of PACS, RIS, and HIS systems [15].

Cross-border referral networks established critical treatment pathways for patients unable to access care locally. Ukrainian medical evacuation programs facilitated transfer of 281 patients from 339 applications to European Union institutions between April 2022 and April 2023, demonstrating systematic coordination of international cancer care [21]. Iraqi patients established cross-border care patterns with Lebanon, with 1,284 registry cases receiving treatment between 2013-2018 [17]. Afghan patients accessed cancer services in Pakistan through the Shaukat Khanum Memorial Cancer Hospital and Research Centre, which accepted patients based on cancer diagnosis without regard to financial status, ethnicity, or nationality, providing free treatment to those unable to pay [18].

Localized service development occurred in some conflict-affected regions. Anbar, Iraq, experienced dramatic expansion of oncology services post-conflict, with oncologist numbers increasing from 1 in 2019 to 11 by 2022, alongside establishment of new oncology centers offering chemotherapy, surgical oncology, and diagnostic services, though radiotherapy remained absent, forcing patients to travel to Baghdad or abroad [19]. Kirkuk’s oncology department expanded from 3 oncologists with 12 beds in 2007 to 5 oncologists with 50-60 beds by 2013, developing sufficient reputation to attract referrals from Baghdad, Nineveh, Diyala, and Tikrit [19].

### Psychosocial and economic impacts

Conflict exacerbated psychosocial burdens for cancer patients beyond the disease itself. Refugee populations reported extremely high rates of adverse mental health conditions including major depression, post-traumatic stress disorder (PTSD), anxiety, and adjustment disorders stemming from violence exposure, traumatic loss of homes/assets, family separation, uncertainty about future, culture shock, and discrimination [15]. Syrian healthcare workers described how exposure to war and worry for safety of loved ones profoundly affected their psychological state, attention, availability, and energy for patient care [3].

Displacement and dislocation from social support networks created additional hardships. Nagorno-Karabakh patients described being away from families and children while staying with friends or in poor-condition hostels in Armenia during treatment, expressing significant discomfort with being outside their comfort zone [1]. Ukrainian cancer patients faced loss of family structure and support with uncertain futures, war-related stress, lack of facilities and infrastructure, shortage of essential medicines and equipment, and financial devastation [13].

Financial toxicity reached catastrophic proportions across conflict settings. Iraqi cross-border patients demonstrated that approximately 33% sold homes and possessions to fund treatment and travel [12]. Northwest Syrian patients in a 2022 survey showed 65% of women did not know where to access mammogram screening, reflecting broader awareness and access gaps [3]. Jordan’s cancer care system, while comprehensive, faced challenges with 35% of health insurance gaps driven by socioeconomic status, with lower rates among unemployed, rural, and refugee populations, resulting in high out-of-pocket expenditures that led to treatment delays, incomplete therapy courses, or treatment abandonment [15].

Hidden costs compounded direct treatment expenses. These included transportation costs for cross-provincial referrals, temporary accommodation expenses in treatment cities, lost income from inability to work during treatment, and caregiver opportunity costs [1, 20]. Nagorno-Karabakh patients described poor living arrangements in temporary hostels provided by the Armenian Government, with inadequate sanitation and proper living conditions [1]. Syrian patients traveling to Turkey for radiotherapy faced waiting times of 1-6 months between referral and treatment initiation, requiring extended stays away from home and livelihoods [3].

### Policy and governance challenges

Post-conflict oncology reconstruction faced significant governance and policy implementation challenges. Political fragmentation following conflict impeded service restoration in some regions. Nineveh and Kirkuk, Iraq, experienced military campaigns against ISIL that fragmented local political order and proliferated competing factions, resulting in lack of governance coherence that slowed reconstruction pace [19]. Kirkuk’s cancer care delivery stagnated amid lack of consensus among competing political factions and reductions in government-provided pharmaceuticals, maintaining only 5 oncologists before, during, and after conflict until 2022 when 3 additional oncologists were added [19].

Conversely, political stability facilitated rapid reconstruction in some settings. Anbar, Iraq, where a single political coalition emerged controlling local government and holding influential federal positions, enabled effective collaboration between the Ministry of Health, Anbar Directorate of Health, and provincial government to restore and expand oncology services. Foreign investment spiking to $11 billion contributed to economic rebuilding, enabling speedy return of both general population and oncology specialists [19].

National policy gaps undermined comprehensive cancer control. Syria lacked a national cancer control plan, with limited screening programs for breast and cervical cancer exclusive to Damascus, highlighting massive inequity in service distribution 3[3]. Yemen demonstrated absence of advanced nuclear diagnostic services such as SPECT, SPECT/CT, or PET/CT, making it the only Arab country without these capabilities [4]. Iraq’s cancer registry and overall data management system suffered under protracted conflict, with war-induced patient displacements and cross-border care-seeking exacerbating data reliability gaps [17].

International humanitarian assistance showed both strengths and limitations. Northwest Syria’s healthcare provision, funded by external donors through NGOs, excluded cancer care from funding as it was classified as tertiary healthcare, forcing NGOs to independently fundraise for cancer services [3]. The blockade of Nagorno-Karabakh prompted Red Cross medical evacuation support, but capacity limitations meant not all patients in need could be evacuated, and justification was required for each request, with patients denied access if they simply presented at checkpoints with medical documentation [1]. Ukrainian pediatric patients received prioritized international evacuation support, with approximately 1,500 children transported via adapted trains to Poland and other European countries, while adult patients had significantly fewer options [21].

## Discussion

### Summary of main findings

In the above healthcare system breakdowns during armed conflicts, the focus has largely been on the most immediate and visible consequences: deaths and injuries from violence, outbreaks of infectious diseases, maternal and child health crises, trauma, and the collapse of primary care systems. Within this framework, cancer has often been neglected and its priority lost compared to other needs, because it is perceived as a chronic condition rather than an acute health emergency.

### Comparison with existing literature

This review corroborates the literature by highlighting the profound and multifaceted impact of armed conflict on cancer care, with consistent patterns of infrastructure destruction, workforce depletion, treatment access barriers, drug supply chain disruptions, and late-stage presentation across diverse settings. The evidence synthesized here, drawn from diverse conflict-affected settings (Palestine, Sudan, Syria, Iraq, Yemen, Afghanistan, Ukraine, Nagorno-Karabakh, Lebanon and others), aligns with themes identified in previous narrative and scoping reviews of in humanitarian crises, but it tackled cancer-specific vulnerabilities that are often under-recognized in humanitarian crises.

The data consistently demonstrate that conflict-affected populations experience higher proportions of late-stage presentation, poorer survival compared with pre-conflict or stable settings. For example, the Syrian lung cancer cohort revealed that nearly two-thirds of patients presented with metastatic disease, with a five-year survival rate approaching zero, figures that are dramatically worse than global averages [20, 27]. In Gaza, it is estimated that the five-year survival rate for Palestinian women diagnosed with breast cancer is only 40% compared to nearly 90% in countries with comprehensive screening and early detection programs [28, 29]. This difference is largely based on the timing of the diagnosis; over 60% of breast cancer cases in Gaza are diagnosed at stage III or later, which is approximately twice that of the US [14, 29].

### Implications for practice and research

Armed conflict does not always cause uniform collapse of oncology care. Government policy may help to develop resilience. In some regions, trajectories differ in timing, magnitude, and adaptation. Caglevic et al. described immediate disruption of cancer centers in Ukraine, prompting urgent evacuation programs [13]. In Syria, patterns were heterogeneous: northern hospitals were destroyed or partially functional with NGO support, while services in Damascus remained largely intact [3, 26]. Yemen saw gradual decline with chemotherapy shortages over months, whereas Gaza experienced abrupt collapse within a month due to blockade-related supply chain disruption and destruction of its only cancer hospital [4, 22]. These differences reflect geography, infrastructure attacks, and presence of evacuation programs. Skelton et al. introduced “therapeutic geographies,” showing how conflict reshapes cancer care across borders, as seen in Iraq’s fragmented transnational networks [12]. Regionally coordinated oncology systems are needed to reduce reliance on ad-hoc cross-border treatment.

To assess the efficacy of adaptation tactics, future studies should go beyond descriptive data. There is an urgent need for prospective studies that assess patient-reported outcomes, financial burden, equity of access, and survival. To guarantee that cancer care is no longer overlooked in crisis response, it is essential to integrate oncology care into humanitarian healthcare.

A narrative commentary also explores how COVID-19 compounded Gaza’s cancer care challenges, calling for global health agencies to support sustainable oncology services in conflict-affected regions [14]. Lessons may apply to other crises, offering ideas for healthcare workers and administrators to aid suffering populations. We sincerely hope this review can help healthcare workers and administrators to brainstorm more ideas to help these suffering countries.

### Limitations

This study is based on English language studies and may miss information from non-English studies. The grey literature is excluded. The lack of information from some countries exactly reflects the real-world situation of their fragmented healthcare system.

## Conclusion

Cancer should be considered a primary issue in humanitarian emergencies. While the immediate focus often centers on trauma and direct injuries, armed conflict imposes sustained and often invisible burdens on cancer patients. These include disrupted access to diagnostics, treatment delays, medication shortages, and the collapse of referral pathways. modified health interventions such as, mobile health units, tele-oncology, and cross-border referral networks have demonstrated that interventions can be effective and achievable. the data points towards a need to reframe cancer as a serious humanitarian health issue, demanding coordinated strategies to ensure continuity of care and safeguard vulnerable populations in crisis settings.

## Supplementary Information

Additional file 1.

## Data Availability

No datasets were generated or analysed during the current study.

## Abbreviations

ASR: Age-standardized rate
CINAHL: Cumulative index to nursing and allied health literature
CIR: Crude incidence rate
COVID-19: Coronavirus disease 2019
CT: Computed tomography
ECOG: Eastern cooperative oncology group performance status
EU: European union
HCAB: Health care access barrier
HIS: Health information system
ISIL: Islamic state of Iraq and the levant
LINAC: Linear accelerator
MedEvac: Medical evacuation
NGO / NGOs: Non-governmental organization(s)
PACS: Picture archiving and communication system
PET/CT: Positron emission tomography / computed tomography
PRISMA: Preferred reporting items for systematic reviews and meta-analyses
PTSD: Post-traumatic stress disorder
RIS: Radiology information systems
SAMS: Syrian american medical society
SPECTSingle: Photon emission computed tomography
SPECT/CT: Single photon emission computed tomography / computed tomography
