# Supplementary material for: Cancer care in conflict-affected regions: a scoping review of service delivery challenges, healthcare system adaptations, and policy implications
Source: Confl Health. 2026 Jun 19;20:76. doi: 10.1186/s13031-026-00816-y (PMC13479888; doi:10.1186/s13031-026-00816-y)
Supplement: Supplementary file 1 [file 13031_2026_816_MOESM1_ESM.docx]

**Preferred Reporting Items for Systematic reviews and Meta-Analyses extension for Scoping Reviews (PRISMA-ScR) Checklist**

| **SECTION** | **ITEM** | **PRISMA-ScR CHECKLIST ITEM** | **REPORTED ON PAGE #** |
| --- | --- | --- | --- |
| **TITLE** | | | |
| Title | 1 | Identify the report as a scoping review. | Title - p1 |
| **ABSTRACT** | | | |
| Structured summary | 2 | Provide a structured summary that includes (as applicable): background, objectives, eligibility criteria, sources of evidence, charting methods, results, and conclusions that relate to the review questions and objectives. | Abstract section – p2 |
| **INTRODUCTION** | | | |
| Rationale | 3 | Describe the rationale for the review in the context of what is already known. Explain why the review questions/objectives lend themselves to a scoping review approach. | Introduction section – p2 |
| Objectives | 4 | Provide an explicit statement of the questions and objectives being addressed with reference to their key elements (e.g., population or participants, concepts, and context) or other relevant key elements used to conceptualize the review questions and/or objectives. | Introduction section – p3 |
| **METHODS** | | | |
| Protocol and registration | 5 | Indicate whether a review protocol exists; state if and where it can be accessed (e.g., a Web address); and if available, provide registration information, including the registration number. | Not reported |
| Eligibility criteria | 6 | Specify characteristics of the sources of evidence used as eligibility criteria (e.g., years considered, language, and publication status), and provide a rationale. | Method section – p4 |
| Information sources* | 7 | Describe all information sources in the search (e.g., databases with dates of coverage and contact with authors to identify additional sources), as well as the date the most recent search was executed. | Method section -p4 |
| Search | 8 | Present the full electronic search strategy for at least 1 database, including any limits used, such that it could be repeated. | Method /search outcome -p5 |
| Selection of sources of evidence† | 9 | State the process for selecting sources of evidence (i.e., screening and eligibility) included in the scoping review. | Method section -p4,5 |
| Data charting process‡ | 10 | Describe the methods of charting data from the included sources of evidence (e.g., calibrated forms or forms that have been tested by the team before their use, and whether data charting was done independently or in duplicate) and any processes for obtaining and confirming data from investigators. | Data extraction – p6-9 |
| Data items | 11 | List and define all variables for which data were sought and any assumptions and simplifications made. | Table 1 on p7-9 |
| Critical appraisal of individual sources of evidence§ | 12 | If done, provide a rationale for conducting a critical appraisal of included sources of evidence; describe the methods used and how this information was used in any data synthesis (if appropriate). | Not conducted |
| Synthesis of results | 13 | Describe the methods of handling and summarizing the data that were charted. | Results section: p9-10 |
| **RESULTS** | | | |
| Selection of sources of evidence | 14 | Give numbers of sources of evidence screened, assessed for eligibility, and included in the review, with reasons for exclusions at each stage, ideally using a flow diagram. | Prisma flow diagram (Figure 1) on p5 |
| Characteristics of sources of evidence | 15 | For each source of evidence, present characteristics for which data were charted and provide the citations. | Table1 on p6-9 |
| Critical appraisal within sources of evidence | 16 | If done, present data on critical appraisal of included sources of evidence (see item 12). | Not applicable |
| Results of individual sources of evidence | 17 | For each included source of evidence, present the relevant data that were charted that relate to the review questions and objectives. | Table 1 and table 2- p6-11 |
| Synthesis of results | 18 | Summarize and/or present the charting results as they relate to the review questions and objectives. | P6-11, 18 |
| **DISCUSSION** | | | |
| Summary of evidence | 19 | Summarize the main results (including an overview of concepts, themes, and types of evidence available), link to the review questions and objectives, and consider the relevance to key groups. | Discussion section -p18 |
| Limitations | 20 | Discuss the limitations of the scoping review process. | Discussion section p20 |
| Conclusions | 21 | Provide a general interpretation of the results with respect to the review questions and objectives, as well as potential implications and/or next steps. | Conclusion section p20 |
| **FUNDING** | | | |
| Funding | 22 | Describe sources of funding for the included sources of evidence, as well as sources of funding for the scoping review. Describe the role of the funders of the scoping review. | Declaration section – funding statement |

**Search Strategy Table**

| **Databases** | **Search string** | **Limits applied** |
| --- | --- | --- |
| **PubMed** | ("cancer care" OR "cancer treatment" OR "oncology services" OR "cancer patients" OR "neoplasms") AND ("conflict-affected" OR "war" OR "armed conflict" OR "conflict zones" OR "humanitarian crisis" OR "fragile states") AND ("service delivery" OR "healthcare system" OR "healthcare access" OR "health system adaptations" OR "policy implications") | English language; Publication date: January 2020–August 2025; Full-text available; Peer-reviewed |
| **Scopus** | TITLE-ABS-KEY ("cancer care" OR "cancer treatment" OR "oncology services" OR "cancer patients") AND TITLE-ABS-KEY ("conflict-affected" OR "war" OR "armed conflict" OR "conflict zones" OR "humanitarian crisis" OR "fragile states") AND TITLE-ABS-KEY ("service delivery" OR "healthcare system" OR "healthcare access" OR "health system adaptations" OR "policy implications") | English language; PUBYEAR > 2019 AND PUBYEAR < 2026; Document type: Article |
| **ScienceDirect** | ("cancer care" OR "cancer treatment" OR "oncology services") AND ("conflict" OR "war" OR "armed conflict" OR "humanitarian crisis" OR "fragile states") AND ("service delivery" OR "healthcare system adaptations" OR "healthcare access" OR "policy implications") | English language; Year: 2020–2025; Article type: Research articles |
| **CINAHL** | ("cancer care" OR "cancer treatment" OR "oncology services" OR "cancer patients") AND ("conflict-affected regions" OR "war" OR "armed conflict" OR "conflict zones" OR "humanitarian crisis" OR "fragile states") AND ("service delivery" OR "healthcare system" OR "healthcare access" OR "health system adaptations" OR "policy implications") | English language; Published Date: January 2020–August 2025; Peer Reviewed; Full Text |
